# In-Hospital Mortality of COVID-19 Patients Treated with High-Flow Nasal Oxygen: Evaluation of Biomarkers and Development of the Novel Risk Score Model CROW-65

**DOI:** 10.3390/life11080735

**Published:** 2021-07-23

**Authors:** Toni Kljakovic Gaspic, Mirela Pavicic Ivelja, Marko Kumric, Andrija Matetic, Nikola Delic, Ivana Vrkic, Josko Bozic

**Affiliations:** 1 Department of Anesthesiology and Intensive Medicine, University Hospital of Split, 21000 Split, Croatia; tkljakgas@kbsplit.hr (T.K.G.); ndelic@kbsplit.hr (N.D.); 2 Department of Infectious Diseases, University Hospital of Split, 21000 Split, Croatia; mpavivelj@kbsplit.hr (M.P.I.); ivvrkic@kbsplit.hr (I.V.); 3 Department of Pathophysiology, University of Split School of Medicine, 21000 Split, Croatia; marko.kumric@mefst.hr; 4 Department of Cardiology, University Hospital of Split, 21000 Split, Croatia; amatetic@kbsplit.hr

**Keywords:** COVID-19, HFNO, in-hospital outcomes, mechanical ventilation, risk scores

## Abstract

To replace mechanical ventilation (MV), which represents the cornerstone therapy in severe COVID-19 cases, high-flow nasal oxygen (HFNO) therapy has recently emerged as a less-invasive therapeutic possibility for those patients. Respecting the risk of MV delay as a result of HFNO use, we aimed to evaluate which parameters could determine the risk of in-hospital mortality in HFNO-treated COVID-19 patients. This single-center cohort study included 102 COVID-19-positive patients treated with HFNO. Standard therapeutic methods and up-to-date protocols were used. Patients who underwent a fatal event (41.2%) were significantly older, mostly male patients, and had higher comorbidity burdens measured by CCI. In a univariate analysis, older age, shorter HFNO duration, ventilator initiation, higher CCI and lower ROX index all emerged as significant predictors of adverse events (*p* < 0.05). Variables were dichotomized and included in the multivariate analysis to define their relative weights in the computed risk score model. Based on this, a risk score model for the prediction of in-hospital mortality in COVID-19 patients treated with HFNO consisting of four variables was defined: CCI > 4, ROX index ≤ 4.11, LDH-to-WBC ratio, age > 65 years (CROW-65). The main purpose of CROW-65 is to address whether HFNO should be initiated in the subgroup of patients with a high risk of in-hospital mortality.

## 1. Introduction

Ever since the COVID-19 pandemic reached its peak in March 2020, intensivists around the world have been struggling to establish the optimal approach for the treatment of its life-threatening complications [1]. Despite the fact that only a small percentage of patients develop severe clinical symptoms, COVID-19 emerged as a major global healthcare issue, because of its potential for rapidly spreading and the large number of people with multiple co-morbidities [2,3,4,5,6]. The COVID-19 pandemic has also had major implications for the global economy, which resulted in harmful repercussions for healthcare systems around the world [7]. This is especially noticeable in poverty-stricken areas, since in those areas, the lack of resources has been a major determinant of disease prognosis since even before the pandemic [7]. Although there is a lot of information on the treatment of severe acute respiratory syndrome (SARS) and Middle East respiratory syndrome (MERS), which are viruses similar to SARS-CoV-2 (the causative agent of COVID-19), there are many differences between COVID-19, SARS and MERS infections [8,9].

So far, the treatment approach for severe cases of COVID-19 has mostly consisted of mechanical ventilation (MV) and corresponding intensive care treatment [10,11]. Indications for intubation still mainly depend on the estimation of the attending clinician, and are very inconsistent among different centers [12,13,14]. Recently, an early intubation approach has also been proposed [15]. This approach has been backed up by multiple studies in patients with acute respiratory distress syndrome (ARDS), conducted in the pre-COVID era [16,17]. However, despite substantial developments in our understanding and managing of the detrimental effects of invasive ventilation, patients whose treatment results in intubation have very poor prognosis, even in the most reputable medical centers [18,19].

High-flow nasal oxygen (HFNO) recently emerged as a less-invasive therapeutic possibility for patients with deteriorating pulmonary function as a consequence of COVID-19 infection [20]. It has been suggested that HFNO can provide high concentrations of humidified oxygen with a low level of positive end-expiratory pressure (PEEP), and facilitate the elimination of CO2, thus alleviating the symptoms of acute hypoxemic respiratory failure (AHRF) [21,22]. Multiple studies have been conducted to this day, both in COVID-19 and non-COVID-19 settings, but the risk of delaying MV induction still remains a major concern for HFNO use in critically ill patients [23,24,25,26,27]. Consequently, there is a need for a reliable risk-stratification model that will facilitate clinical decision -aking by predicting the risk of failure of the HFNO therapy.

In this study, we reported the outcomes of in-hospital mortality for patients with COVID-19 treated with HFNO in our tertiary hospital center. Moreover, we aimed to evaluate which parameters could determine the risk of in-hospital mortality in HFNO-treated COVID-19 patients. Based on our findings, we proposed a risk score model consisting of four variables, which combines anamnestic and laboratory data.

## 2. Materials and Methods

### 2.1. Study Design and Ethical Considerations

**Figure 1:**
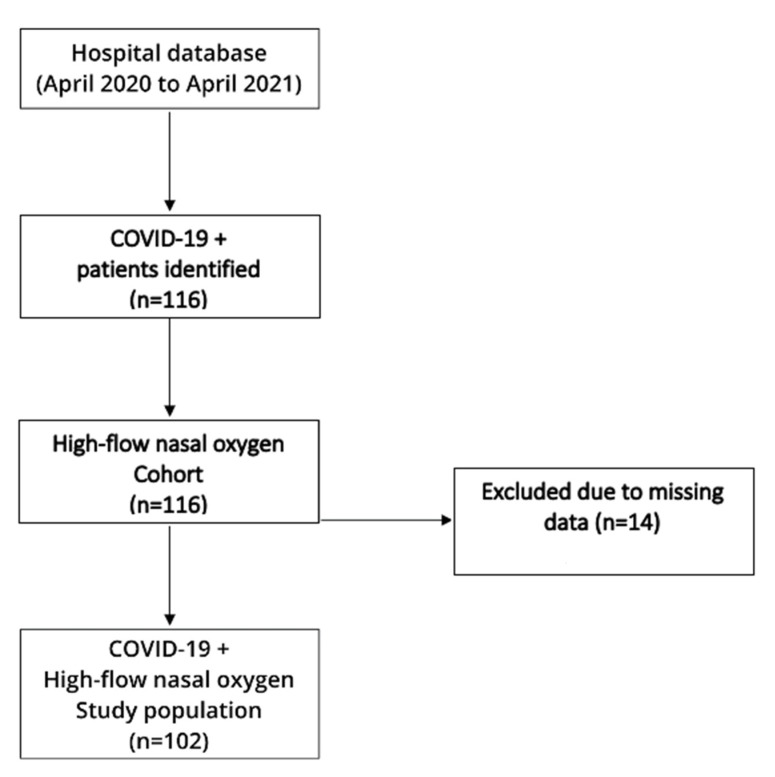
Flow diagram of the study. COVID-19: coronavirus disease 2019.

This single-center cohort study was conducted at the University Hospital of Split Respiratory Intensivist Center (RIC) from April 2020 to April 2021. The study was approved by the Ethics Committee of the University Hospital of Split (Class: 500-03/21-01/02. Number: 2181-147-01/06/M.S.-20-02) and was conducted in accordance with all ethical principles of the Declaration of Helsinki, 2013. The flow diagram of the study is depicted in Figure 1.

### 2.2. Subjects and Inclusion/Exclusion Criteria

The present study included 102 polymerase chain reaction confirmed SARS-CoV-2 positive adult patients (≥18 years) with hypoxemic respiratory failure, treated with HFNO for ≥2 h, at the RIC of the University Hospital of Split. Exclusion criteria were as follows: patients with dementia, terminal stage of malignant disease, uncooperative patients, acute hypercapnic respiratory failure and missing data. We initially included 116 patients in our study, but 14 patients were excluded due to missing data.

### 2.3. Clinical and Laboratory Evaluations

Data on demographic characteristics (age, sex) and history of chronic diseases (hypertension, diabetes, cerebrovascular disease, chronic obstructive pulmonary disease, malignant tumors and other chronic diseases) were collected from the hospital records for each included patient. Vital signs and SpO2 were continuously monitored upon admission to the RIC by an attending physician. We used the Charlson Comorbidity index (CCI), which predicts 10-year survival in patients with multiple comorbidities, for the assessment of comorbidity burden. The main variables of the CCI are age and the following diseases, based on which points are awarded: myocardial infarction, heart failure, peripheral vascular disease, cerebrovascular insult, dementia, COPD, connective tissue disease, peptic ulcer disease, diabetes mellitus, hemiplegia, chronic kidney disease, solid tumor, leukemia, lymphoma and AIDS. In the present study, we used the ROX index, calculated by the ratio of SpO2/FiO2 to the respiratory rate.

Arterial blood gas variables (pH, PaCO2, PaO2, bicarbonate) and laboratory tests (complete blood count, white blood cells (WBCs), C-reactive protein (CRP), lactate dehydrogenase (LDH), and D-dimer) were measured on the day of admission to the RIC. In addition, arterial blood gas variables were collected each day, 3 times per day or more depending on patient’s respiratory condition. We collected these values on the first and the last days of the HFNO therapy. All laboratory analyses were performed in the same biochemical laboratory and measured by standard laboratory methods.

HFNO was indicated after standard oxygen therapy (nasal cannula, reservoir mask) failure. We used a humidifier with an integrated flow generator that delivers high flow warmed and humidified respiratory gases through the nose to spontaneously breathing patients (HFNO, Airvo2TM, Fisher and Paykel Healthcare, Auckland, New Zealand). High-flow nasal oxygen treatment was initiated when SpO2 and PaO2/FiO2 (P/F ratio) were low even after maximum oxygen flow (15 L/min with reservoir mask) was reached. HFNO failure was defined as upgrading respiratory support to MV or death after HFNO treatment. The standard references for endotracheal intubation include the following: airway protection, severe decompensate acidosis (pH < 7.2), and severe absolute hypoxemia (PaO2 < 50 mmHg or SpO2 < 90%) despite maximal noninvasive respiratory support (HFNO).

The following standard therapeutic methods and up-to-date protocols were used in patient treatment: corticosteroids, antiviral medications, anticoagulants, oxygen, and other supportive therapies.

### 2.4. Statistical Analysis

Statistical analysis was conducted according to standard statistical methods. The normality of data distribution was assessed by Kolmogorov–Smirnov test. Categorical variables were reported as numbers (percentages) and compared using the chi-squared test, while continuous data were expressed as the mean ± standard deviation (SD) or as the median (interquartile range, IQR), and were compared using Student’s T-test or the Mann–Whitney U test, respectively. Univariate Cox logistic regression analysis with the enter algorithm was performed to determine the predictors of in-hospital mortality. The results of the risk analyses were expressed as the hazard ratio (HR) with 95% confidence intervals (95% CI). Single variables that were found to be significant predictors (*p* < 0.05) in a univariate analysis were used to compute a risk score model in a multivariate analysis. Risk score modeling was based on the regression coefficients of categorical (dichotomized) variables, according to the recommendations [28]. A regression coefficient-based scoring system was used, as it has shown better performance in data fitting. Furthermore, the number of predictor-variables was carefully selected to ensure at least 10 events per predictor-variable according to the “rule of thumb”. Rounding and scaling of the coefficients was performed to determine the contribution of each variable to the final score. The Youden index was used to define the optimal cut-off value with the best sensitivity and specificity ratio according to the Henley and McNeil method. Internal validation of the risk score model was conducted by confirming its predictive accuracy with receiver operating characteristic (ROC) analysis and evaluating mortality rates across different risk score tertiles. Furthermore, the accuracy of each biomarker in predicting in-hospital mortality was tested using ROC analysis, with a calculation of area under the curve (AUC). The cumulative incidence of in-hospital mortality was estimated using the Kaplan–Meier approach, and significance was assessed using the Mantel–Cox log-rank test. A two-sided *p*-value of <0.05 was considered significant. Statistical data analysis was carried out using the Statistical Package for the Social Sciences (SPSS) software (IBM Corp, NY, USA; version 20).

## 3. Results

The study population mostly consisted of older adult male patients (median of 66 years and 71.6%, *n* = 73, respectively). Of the 102 enrolled patients, death occurred in 42 patients (41.2%). Patients who suffered fatal outcomes were significantly older, mostly men, and had higher comorbidity burdens as measured by CCI. Additionally, these patients exhibited lower values of ROX index and hemoglobin, and had shorter durations of HFNO therapy with a higher prevalence of ventilator initiation. There were no statistically significant differences in other anthropometric characteristics, comorbidities, or laboratory parameters (Table 1). 

There was no statistically significant difference in the values of different biomarkers between groups, except in the values of the hemoglobin-to-RDW ratio, which was significantly lower (9.42 ± 2.01 vs. 10.15 ± 1.48, *p* = 0.037), and the LDH-to-WBC ratio, which was significantly higher (79.44 ± 7.36 vs. 54.55 ± 5.65, *p* = 0.022), in patients who died (Table 2).

When evaluating different predictors of in-hospital mortality, older age, shorter HFNO duration, ventilator initiation, higher CCI and lower ROX index were all significant predictors of adverse events (*p* < 0.05) (Table 3). 

**Figure 2:**
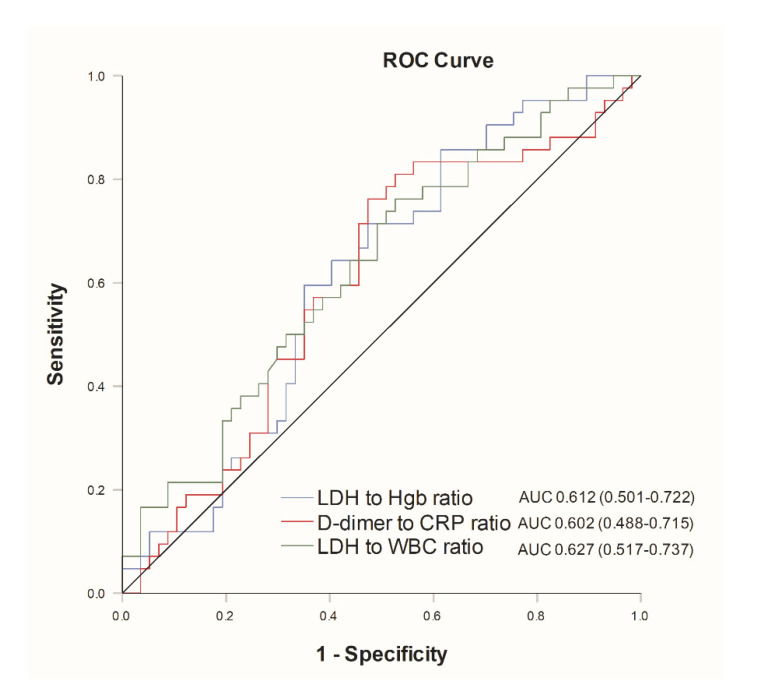
Receiver-operator characteristics analysis of selected biomarkers for in-hospital mortality. Abbreviations: AUC: area under the curve; CRP: C-reactive peptide; Hgb: hemoglobin; LDH: lactate dehydrogenase; WBC: white blood cells.

There was significant difference in mortality between groups depending on ventilator initiation. Patients who required MV had significantly higher in-hospital mortality (76.2% vs. 15.5%). In the univariate Cox regression analysis, CRP-to-lymphocyte ratio proved to be a significant predictor of in-hospital mortality, whereas LDH-to-WBC ratio, LDH-to-hemoglobin ratio and D-dimer-to-CRP ratio had the best AUC values (0.627, 0.612 and 0.602 respectively), i.e., the best accuracy in predicting in-hospital mortality (Figure 2).

Variables that were found to be independent predictors of in-hospital mortality in the univariate analysis were dichotomized and included in the multivariate analysis to define the relative weights of each variable in the computed risk score model (Table 4).

**Figure 3:**
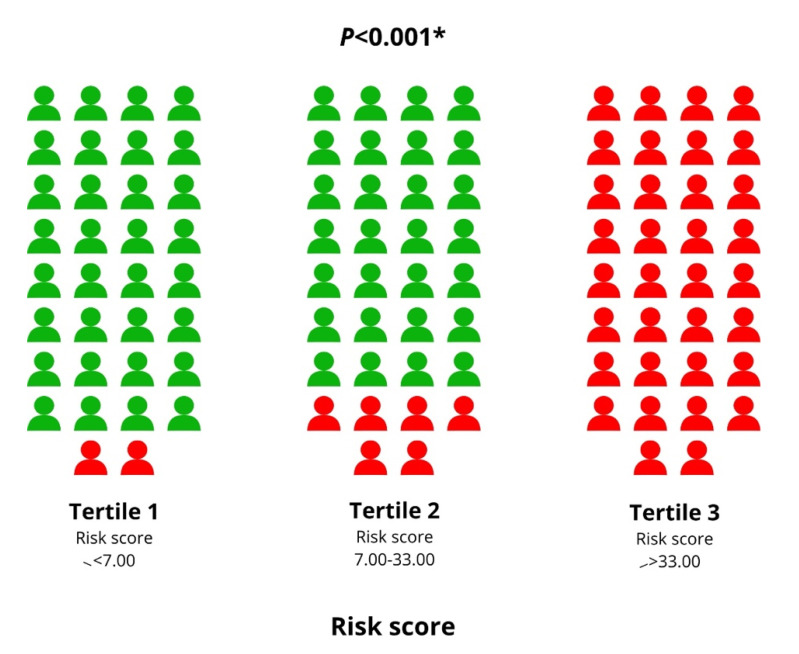
Evaluation of in-hospital mortality across different risk score tertiles. Red color indicates patients with in-hospital mortality, whereas the green represents survivors. * Chi squared test.

**Figure 4:**
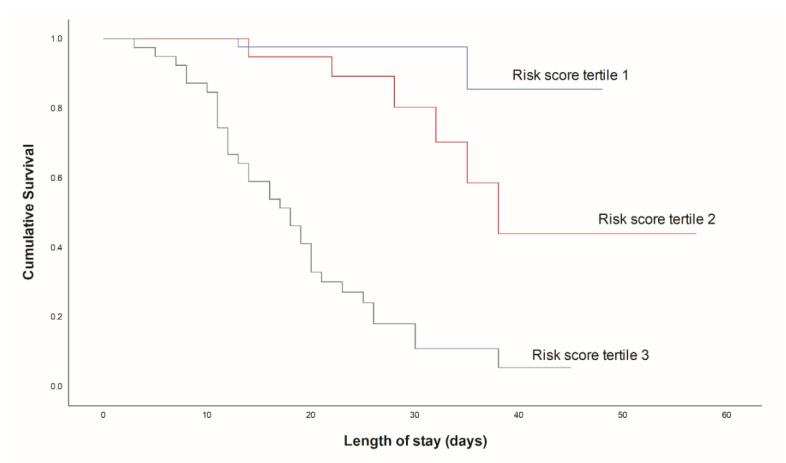
Cumulative survival of groups based on risk score tertiles.

**Figure 5:**
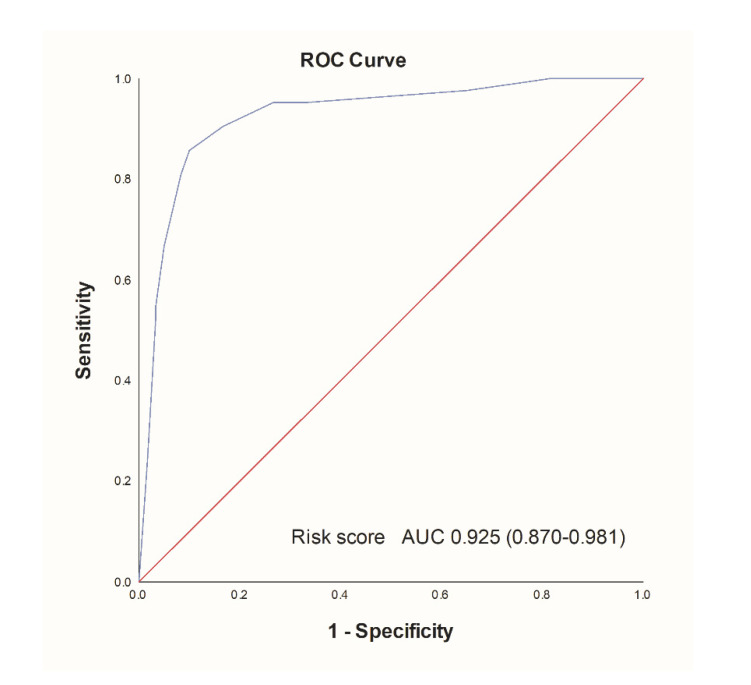
Receiver-operator characteristics analysis of the risk score model for in-hospital mortality. Abbreviations: AUC: area under the curve.

Finally, a risk score model consisting of four variables was defined: CCI > 4, ROX index ≤ 4.11, LDH-to-WBC ratio, age > 65 years (CROW-65) (Table 5). It proved to have satisfying stratification strength, showing the lowest mortality rates in the lowest risk score tertile (*n* = 2, 5.9%), followed by the second tertile (*n* = 6, 17.6%) and the third tertile (*n* = 34, 100%; *p* < 0.001) (Figure 3). Similarly, the cumulative incidence of mortality was significantly different between risk score tertiles, with the highest risk score tertile having the highest in-hospital mortality (*p* < 0.001) (Figure 4). Finally, the risk score model showed significant accuracy in predicting in-hospital mortality, with an AUC of 0.925 (0.870–0.981, *p* < 0.001) (Figure 5).

## 4. Discussion

In the present study, COVID-19 patients treated with HFNO who died in-hospital were significantly older, mostly male patients, with higher comorbidity burdens and a higher prevalence of ventilator initiation, while exhibiting lower ROX index, hemoglobin levels, and duration of HFNO therapy. Based on a multivariate analysis, we created the CROW-65, a risk score model for the prediction of in-hospital mortality consisting of four variables: CCI > 4, ROX index ≤ 4.11, LDH-to-WBC ratio, age > 65 years. This risk score model showed significant accuracy in predicting the in-hospital mortality of COVID-19 patients treated with HFNO. To the best of our knowledge, this is the first study to use this risk score model for in-hospital mortality prediction.

Early into the onset of the COVID-19 pandemic, the HFNO emerged as a bridging supportive modality for the management of patients with severe COVID-19 [29,30,31,32,33,34], primarily based on the available data for patients with severe pneumonia in the pre-COVID era [21,35,36,37,38]. The rate of HFNO failure in our study (40.2%) is similar to the failure rates of the above-noted studies. The biggest question with respect to HFNO use in this setting was the establishment of appropriate timing for MV induction. A doubt was raised that prolonging HFNO therapy could in certain patients delay the inevitable need for MV, thus jeopardizing their clinical outcomes [29]. However, in a study by Hu et al., the authors demonstrated that postponing MV with HFNO did not substantially contribute to mortality burden, although mortality rates in patients requiring MV after HFNO failure were overall high (78.5%), whereas Chandel et al. did not find any difference between patients with early (<48 h) and late (>48 h) HFNO failure [30,31]. On the other hand, Duan et al. demonstrated that patients treated with HFNO in resource-limited areas experience a longer duration of hypoxemia and delayed escalation care, which possibly resulted in the higher mortality rates observed in those areas, thus highlighting the importance of timely transfer from HFNO to MV [34]. Finally, as discussed by multiple authors, the crucial step to avoid a delay in escalation therapy in COVID-19 patients is intensive monitoring during HFNO therapy [33,39]. In terms of mortality, our cohort of patients in which HFNO failed yielded similar results as the aforementioned study by Hu et al. [32]. However, other studies reported much lower mortality rates among their patients [31,34,40]. The observed difference is probably owing to the discrepancy in clinical characteristics of treated patients, primarily the severity of the disease. Namely, the ROX index at the time of hospital admission, a valuable indicator of poor outcomes in patients with AHRF, was substantially lower in our and Hu’s study in comparison to the studies with lower mortality rates [32,33,40,41,42]. Furthermore, during the time in which the study was conducted, the British variant of SARS-CoV-2 breached our department, thus yielding less favorable outcomes. In line with this, results from the present study, and other studies as well, suggest a strong association between ROX index, calculated by the ratio of SpO2/FiO2 to the respiratory rate, and in-hospital mortality from COVID-19 [31,33,43]. Therefore, taking into consideration these results and the fact that ROX is easily obtained at the bedside, we included it in the risk score model of the present study.

Our results, which imply that higher CCI and advanced age predict in-hospital mortality, are concordant with the available data, as so far it has been well established that aging patients with multiple comorbidities are more prone to the development of severe forms of COVID-19, resulting in poorer outcomes [44,45,46]. 

Among the multiple biomarker ratios we measured, LDH-to-WBC ratio attracted the most attention, as it exhibited the most favorable AUC values while evaluating accuracy in predicting in-hospital mortality. These results are discordant with those of Eckel et al. [47]. Namely, Eckel et al. established that although it is a useful diagnostic predictor, LDH-to-WBC ratio failed to predict severe courses of COVID-19. The observed discrepancy could be due to the differences in the studied population, as we included only patients who required HFNO, whereas Eckel et al. included all patients with documented results of a SARS-CoV-2 PCR assay regardless of the disease severity. Conversely, CRP-to-lymphocyte ratio predicted severe outcomes in the same study, yet in ours there was no significant correlation with in-hospital mortality [47]. Of note, unlike the study by Yang et al., in which neutrophile-to-lymphocyte ratio (NLR) was the best independent predictor for poor clinical outcomes in COVID-19 with an AUC of 0.84, our data suggest that there is no significant correlation between NLR values and in-hospital mortality [48].

By combining the aforementioned parameters, which demonstrated independent prediction strengths (age, ROX index, LDH-to-WBC ratio and CCI), we developed an in-hospital mortality risk score model. The purpose of this scoring system is to determine which patients are at high risk of HFNO failure. Subsequently, clinicians would be more prone to induce MV early in patients with higher scores, thus alleviating the detrimental effects that could arise from the MV delay. This personalized approach is vital for optimal outcomes and, in the future, it could facilitate clinical decisions in COVID-19, as well as in other diseases that lead to AHRF and a consequent need for HFNO.

Other authors reported prognostic risk-stratification models as well. Xu et al. combined age, ROX index, platelet count and interleukin 6 (IL-6) at HFNO initiation, exhibiting a sensitivity of 80.3% and a specificity of 71.2%, and a better predictive strength than ROX index [33]. Although it is valuable, the main setback of this score is the accessibility of IL-6 in areas with limited resources. Mellado-Artigas et al. derived a cheap yet reliable prognostic model that consists of the Baseline Non-respiratory Sequential Organ Failure Assessment score and ROX index [23]. Nonetheless, it is important to point out that most authors used ROX index exclusively in the prediction of HFNO failure.

The main limitation of the present study is its single-center design, as well as the limited number of patients included. The low sample size of this study could lead to imprecise risk predictions or model overfitting, which may result in over-optimistic model performance within the development dataset and poor model performance outside of the development dataset. However, it is difficult to assess whether increasing the sample size will improve model performance, given that model performance is affected by many other factors (prevalence of outcome, inclusion of important predictors, strength of association between predictors and outcome, etc.). Moreover, the authors aimed to respect the “rule of thumb” for effective sample sizes and to ensure at least 10 events per predictor-variable. Furthermore, the prognostic model we have provided has yet to be validated in other, more heterogenous populations. Nevertheless, the authors aim to facilitate future studies with the aim of validating the constructed risk score.

## 5. Conclusions

In summary, the rates of HFNO failure and mortality were generally high in this single-center study, but concordant with most of the available data. Among different parameters, CCI, ROX index, LDH-to-WBC ratio, and age > 65 years resulted in the best prediction of in-hospital mortality. Hence, using these parameters, we established a risk score, the CROW-65, for the prediction of in-hospital mortality in COVID-19 patients treated with HFNO. The main purpose of CROW-65 is to facilitate clinical decision-making in this setting. Specifically, the score could help to determine whether HFNO should be initiated in high-risk patients prone to adverse outcomes. The suggested tailored approach could potentially improve the outcomes of these patients, since multiple studies suggest that delaying MV induction could result in poorer outcomes in severe COVID-19 patients. However, further large-scale studies are warranted to support these notions.

## Figures and Tables

**Table 1:** Comparison of baseline characteristics.

Variables	Death Event		Total (*n* = 102)	*p* Value
	No (*n* = 60)	Yes (*n* = 42)		
Age (years)	63 (57–72)	71 (66–77)	66 (58–73)	<0.001 *
Male sex	40 (66.7%)	33 (78.6%)	73 (71.6%)	0.190 †
Disease duration at admission (days)	9 (8–11)	10 (7–11)	9 (8–11)	0.415 *
ROX index	5.25 ± 1.26	2.90 ± 1.00	4.27 ± 1.64	<0.001 ‡
HFNO duration (days)	9 (5–12)	5 (4–8)	7 (4–11)	0.005 *
Disease duration at HFNO interruption (days)	20 (16–24)	18 (16–23)	19 (16–24)	0.283 *
Corticosteroid therapy (%)	60 (100.0%)	42 (100.0%)	102 (100.0%)	/
Remdesivir therapy (%)	57 (95.0%)	35 (83.3%)	92 (90.2%)	0.051 †
Ventilator (%)	9 (15.5%)	32 (76.2%)	41 (40.2%)	<0.001 †
Comorbidities				
Active smoking	32 (53.3%)	21 (50.0%)	53 (52.0%)	0.740 †
Obesity	5 (8.3%)	4 (9.5%)	9 (8.8%)	0.835 †
Arterial hypertension	37 (61.7%)	28 (66.7%)	65 (63.7%)	0.605 †
Diabetes mellitus	19 (31.7%)	8 (19.0%)	27 (26.5%)	0.155 †
COPD	1 (1.7%)	4 (9.5%)	5 (4.9%)	0.070 †
CCI	2.43 ± 1.14	3.55 ± 1.95	2.89 ± 1.62	<0.001 ‡
Laboratory parameters				
SpO_2_ (%)	81.95 ± 10.84	80.61 ± 11.57	81.40 ± 11.11	0.552 ‡
pH (units)	7.44 ± 0.06	7.43 ± 0.05	7.44 ± 0.06	0.694 ‡
pO_2_ (kPa)	6.15 ± 1.21	6.36 ± 1.31	6.23 ± 1.25	0.418 ‡
pCO_2_ (kPa)	4.35 ± 0.47	4.43 ± 0.65	4.38 ± 0.55	0.437 ‡
HCO_3_^−^ (mmol/L)	25.85 ± 2.49	25.23 ± 3.31	25.60 ± 2.86	0.284 ‡
Hemoglobin (g/L)	140.57 ± 14.96	134.26 ± 15.66	137.97 ± 15.49	0.042 ‡
RDW (%)	13.99 ± 1.43	16.85 ± 18.09	15.17 ± 11.66	0.226 ‡
Platelets (x10^9^/L)	236 (171–317)	253 (165–311)	250 (169–314)	0.497 *
WBC (x10^9^/L)	10.78 ± 5.28	9.10 ± 4.29	10.09 ± 4.94	0.090 ‡
Neutrophiles (%)	81.99 ± 10.63	83.57 ± 8.49	82.64 ± 9.79	0.424 ‡
Lymphocytes (%)	12.08 ± 9.39	10.84 ± 7.69	11.57 ± 8.71	0.482 ‡
Monocytes (%)	4.52 ± 1.91	4.89 ± 1.96	4.67 ± 1.93	0.344 ‡
Eosinophiles (%)	0.29 ± 0.33	0.40 ± 0.53	0.33 ± 0.42	0.221 ‡
CRP (mmol/L)	152 (82–251)	128 (90–199)	146 (85–214)	0.321 *
LDH (umol/L)	451 (345–676)	454 (387–614)	453 (372–659)	0.240 *
D-dimers (mmol/L)	2 (1–4)	4 (1–6)	2 (1–5)	0.449 *

Data are expressed as mean ± SD, number (percent) or median (interquartile range). * Mann–Whitney U test; † Chi-square test; ‡ Student’s *T*-test. Abbreviations: CCI: Charlson comorbidity index; COPD: chronic obstructive pulmonary disease; CRP: C-reactive peptide; HCO_3_^−^: bicarbonate; HFNO: high-flow nasal oxygen; LDH: lactate dehydrogenase; ROX: respiratory rate–oxygenation index; pO_2_: partial pressure of oxygen in the blood; pCO_2_: partial pressure of carbon dioxide in the blood; RDW: red cell distribution width; SpO_2_: oxygen saturation; WBC: white blood cells.

**Table 2:** Comparison of different biomarker ratios.

Variables	Death Event		Total (*n* = 102)	*p* Value
	No (*n* = 60)	Yes (*n* = 42)		
NLR ratio	9 (5–16)	9 (6–17)	9 (6–16)	0.285 *
Hemoglobin to RDW ratio	10.15 ± 1.48	9.42 ± 2.01	9.85 ± 1.75	0.037 †
LDH to hemoglobin ratio	3 (2–5)	4 (3–5)	3 (3–5)	0.118 *
Eosinophile to lymphocyte ratio	0.04 ± 0.06	0.10 ± 0.33	0.07 ± 0.22	0.166 †
CRP to lymphocyte ratio	29.36 ± 4.99	42.56 ± 7.33	34.79 ± 8.06	0.459 †
D-dimer to CRP ratio	0.06 ± 0.19	0.05 ± 0.07	0.06 ± 0.15	0.621 †
LDH to WBC ratio	54.55 ± 5.65	79.44 ± 7.36	64.80 ± 5.50	0.022 †

Data are expressed as mean±SD, number (percent) or median (interquartile range).* Mann–Whitney U test; † Student’s *T*-test. Abbreviations: CRP: C-reactive peptide; LDH: lactate dehydrogenase; NLR: neutrophile to lymphocyte ratio; RDW: red cell distribution width; WBC: white blood cells.

**Table 3:** Univariate Cox regression analysis of different factors for in-hospital mortality.

Variables	HR (95% CI)	*p* Value *
Age	1.04 (1.01–1.08)	0.006
Male sex	1.50 (0.71–3.14)	0.285
Disease duration at admission	1.08 (0.98–1.19)	0.106
Disease duration at HFNO initiation	1.03 (0.97–1.10)	0.324
HFNO duration	0.89 (0.83–0.96)	0.003
Remdesivir treatment	0.53 (0.23–1.19)	0.122
Ventilator initiation	5.74 (2.81–11.69)	<0.001
CCI	1.27 (1.12–1.45)	<0.001
ROX index	0.46 (0.35–0.60)	<0.001
pO_2_	1.15 (0.90–1.48)	0.274
pCO_2_	1.32 (0.78–2.23)	0.305
HCO_3_^−^	0.96 (0.87–1.05)	0.370
Hemoglobin	0.98 (0.96–1.01)	0.080
RDW	1.01 (0.99–1.03)	0.108
Platelets	1.00 (0.99–1.01)	0.229
WBC	0.93 (0.87–1.00)	0.055
Neutrophiles	0.93 (0.86–1.00)	0.061
Lymphocytes	0.74 (0.39–1.41)	0.359
Monocytes	0.93 (0.32–2.72)	0.900
CRP	1.00 (0.99–1.00)	0.162
LDH	0.99 (0.98–1.01)	0.866
D-dimers	1.00 (0.97–1.03)	0.988

Data are expressed as hazard ratios (95% confidence interval). * Cox regression analysis. Abbreviations: CCI: Charlson comorbidity index; CI: confidence interval; CRP: C-reactive peptide; HFNO: high-flow nasal oxygen; HR: hazard ratios; LDH: lactate dehydrogenase; ROX: respiratory rate–oxygenation index.

**Table 4:** Receiver-operator characteristics analysis of selected factors for in-hospital mortality (Henley and McNeil method).

Variables	Youden Index	Sensitivity/Specificity	AUC (95% CI)	*p* Value *
Age	≤65	65.0%/76.2%	0.705 (0.606–0.791)	<0.001
LDH-to-WBC ratio	≤42.75	48.33%/76.19%	0.633 (0.532–0.726)	0.017
CCI	≤3	83.3%/40.5%	0.673 (0.573–0.763)	0.002
ROX index	>4.12	81.4%/92.9%	0.892 (0.814–0.945)	<0.001

Data are expressed as hazard ratios (95% confidence interval). * Cox regression analysis. Abbreviations: CI: confidence interval; CRP: C-reactive peptide; HR: hazard ratios; LDH: lactate dehydrogenase; NLR: neutrophile to lymphocyte ratio; RDW: red cell distribution width; WBC: white blood cells.

**Table 5:** Risk score modeling—CROW-65 risk score.

Variables	Score
CCI >4	3
ROX index ≤ 4.11	26
LDH-to-WBC ratio	7
Age > 65 years	5
Total	41

Abbreviations: CCI: Charlson comorbidity index; CI: confidence interval; HFNO: high-flow nasal oxygen; HR: hazard ratios; ROX: respiratory rate–oxygenation index; LDH: lactate dehydrogenase; WBC: white blood cells.

## Data Availability

The data presented in this study are available on request from the corresponding author. The data are not publicly available because some of the data set will be used for further research.
